# The age of evidence-based orthopaedics

**DOI:** 10.4103/0019-5413.40243

**Published:** 2008

**Authors:** Mohit Bhandari, Paul Tornetta III

**Affiliations:** Department of Orthopaedic Surgery, McMaster University, Hamilton, Ontario, Canada; * Department of Orthopaedic Surgery, Boston University, Boston, USA

Symposium: A Practical Approach to Evidence-Based Orthopaedics

*The process of scientific discovery is, in effect, a continual flight from wonder*.

Albert Einstein

Evidence-based medicine (EBM) was recently noted as one of the top 15 medical discoveries over the past 160 years. The term EBM has become so commonplace in the practice of medicine that it seems hard to believe that the term, itself, was only coined in 1990 at McMaster University (Canada). The popularity of EBM has only recently expanded to evidence-based orthopedics. In 2003, the *Journal of Bone and Joint Surgery* adopted EBM and levels of evidence grading for all clinical papers. Since then, all major North American orthopedic meetings and journals have adopted evidence-based orthopedic teachings in some format or another.

Evidence-based orthopedics requires an understanding of a new language and principle in the evaluation of clinical research and its impact on patient decision-making. While the concepts may not be entirely intuitive, they are an absolute prerequisite to any surgeon who wishes to practice in the era of EBM. Educational efforts have largely focused on medicine with little focus on the unique educational needs of surgeons. The unique features of surgical research and the associated challenges have often been minimized by researchers and practitioners promoting double randomized controlled drug trials as the gold standard.

The current symposium has been developed to cover practical concepts of evidence-based orthopedics including its history, definitions, quality of the literature, checklists for surgeons to evaluate orthopedic literature and communication with patients.

The core articles were developed by researchers at McMaster University in collaboration with experts from Boston (Massachussetts), Toronto (Ontario), Greenville (South Carolina), Zurich (Switzerland), Kiel (Germany), New Delhi (India) and Pune (India).

